# A Dutch highly pathogenic H5N6 avian influenza virus showed remarkable tropism for extra-respiratory organs and caused severe disease but was not transmissible via air in the ferret model

**DOI:** 10.1128/msphere.00200-23

**Published:** 2023-07-10

**Authors:** Sander Herfst, Lineke Begeman, Monique I. Spronken, Marjolein J. Poen, Dirk Eggink, Dennis de Meulder, Pascal Lexmond, Theo M. Bestebroer, Marion P. G. Koopmans, Thijs Kuiken, Mathilde Richard, Ron A. M. Fouchier

**Affiliations:** 1 Department of Viroscience, Erasmus University Medical Center, Rotterdam, the Netherlands; 2 Academic Medical Center Amsterdam, Laboratory of Experimental Virology, Amsterdam, the Netherlands; Emory University School of Medicine, Atlanta, Georgia, USA

**Keywords:** influenza, HPAI, H5N6, transmission, risk assessment, ferret, pathogenesis

## Abstract

Continued circulation of A/H5N1 influenza viruses of the A/goose/Guangdong/1/96 lineage in poultry has resulted in the diversification in multiple genetic and antigenic clades. Since 2009, clade 2.3.4.4 hemagglutinin (HA) containing viruses harboring the internal and neuraminidase (NA) genes of other avian influenza A viruses have been detected. As a result, various HA-NA combinations, such as A/H5N1, A/H5N2, A/H5N3, A/H5N5, A/H5N6, and A/H5N8 have been identified. As of January 2023, 83 humans have been infected with A/H5N6 viruses, thereby posing an apparent risk for public health. Here, as part of a risk assessment, the *in vitro* and *in vivo* characterization of A/H5N6 A/black-headed gull/Netherlands/29/2017 is described. This A/H5N6 virus was not transmitted between ferrets via the air but was of unexpectedly high pathogenicity compared to other described A/H5N6 viruses. The virus replicated and caused severe lesions not only in respiratory tissues but also in multiple extra-respiratory tissues, including brain, liver, pancreas, spleen, lymph nodes, and adrenal gland. Sequence analyses demonstrated that the well-known mammalian adaptation substitution D701N was positively selected in almost all ferrets. In the *in vitro* experiments, no other known viral phenotypic properties associated with mammalian adaptation or increased pathogenicity were identified. The lack of transmission via the air and the absence of mammalian adaptation markers suggest that the public health risk of this virus is low. The high pathogenicity of this virus in ferrets could not be explained by the known mammalian pathogenicity factors and should be further studied.

**IMPORTANCE:**

Avian influenza A/H5 viruses can cross the species barrier and infect humans. These infections can have a fatal outcome, but fortunately these influenza A/H5 viruses do not spread between humans. However, the extensive circulation and reassortment of A/H5N6 viruses in poultry and wild birds warrant risk assessments of circulating strains. Here an in-depth characterization of the properties of an avian A/H5N6 influenza virus isolated from a black-headed gull in the Netherlands was performed *in vitro* and *in vivo*, in ferrets. The virus was not transmissible via the air but caused severe disease and spread to extra-respiratory organs. Apart from the detection in ferrets of a mutation that increased virus replication, no other mammalian adaptation phenotypes were identified. Our results suggest that the risk of this avian A/H5N6 virus for public health is low. The underlying reasons for the high pathogenicity of this virus are unexplained and should be further studied.

## INTRODUCTION

Highly pathogenic avian influenza (HPAI) A/H5 viruses of the A/goose/Guangdong/1/1996 (GsGd) lineage first emerged in Hong Kong in 1997 (1). Since 2003, these viruses have spread throughout Eurasia, Africa, and the Americas. As a result of continuous circulation in poultry, A/H5 GsGd viruses have diversified into numerous distinct genetic and antigenic (sub)clades, and new clades continue to emerge and circulate. The genetic diversity of A/H5 GsGd viruses is further increased by reassortment of viruses carrying the GsGd hemagglutinin (HA) with co-circulating low pathogenic avian influenza (LPAI) viruses from domestic and wild birds. In particular, the A/H5 HA of clade 2.3.4.4 has reassorted frequently with neuraminidase (NA) genes of other subtypes than N1, such as N2, N3, N5, N6, and N8 (2, 3). In 2007, an international group of scientists and collaborators, referred to as the WHO/OIE/FAO H5N1 Evolution Working Group, was created to develop a unified nomenclature of H5 GsGd viruses and to provide regular updates on their genetic diversification (4). So far, clade 2.3.4.4 HAs have been classified in eight subclades (2.3.4.4a-h).

Clade 2.3.4.4 A/H5N6 viruses were first detected in China in a live poultry market in 2013 (5). HPAI A/H5N6 viruses have subsequently been detected in wild birds and poultry in China, Vietnam, and Laos since 2014, in Hong Kong since 2015, and in Japan and South Korea since 2016 (6 - 8). By late 2017, A/H5N6 was also detected in Myanmar (9) and Taiwan (10). Since 2014, multiple other genotypes have also emerged upon reassortment with circulating A/H9N2 or A/H7N9 viruses, resulting in the identification of multiple distinct genotypes (11). As of March 2023, 84 laboratory-confirmed cases of human infection with A/H5N6 viruses have been reported since 2014, including 33 deaths (https://www.who.int/docs/default-source/wpro---documents/emergency/surveillance/avian-influenza/ai_20230331.pdf?sfvrsn=5bc7c406_23).

The first European detection of an HPAI A/H5N6 virus, carrying the clade 2.3.4.4b HA, was detected in a duck farm in the Netherlands in December 2017. The virus was subsequently detected in wild birds in close proximity to the infected farm (12) and in two other commercial poultry holdings. Genetically similar viruses were subsequently detected in the beginning of 2018 in commercial farms and wild birds in Switzerland, the United Kingdom, Germany, Sweden, Ireland, Denmark, Slovak Republic, and Finland (https://food.ec.europa.eu/animals/animal-diseases/diseases-and-control-measures/avian-influenza_en).

Several research groups previously reported on the relatively low pathogenicity and lack of airborne transmissibility of the A/H5N8 clade 2.3.4.4a and clade 2.3.4.4b viruses in the ferret model (13 - 16). Several A/H5N6 viruses were also found to have low pathogenicity in ferrets, although Asian A/H5N6 viruses were shown to be more pathogenic in ferrets and replicated to higher titers in the respiratory tract (17 - 23). Here, we focused our investigation on the European A/H5N6 virus A/black-headed gull/Netherlands/29/2017 (A/H5N6 BHG), which was isolated from a black-headed gull (*Chroicocephalus ridibundus*) on 18 December 2017 (12) as part of a surveillance program (24). *In vitro* characterization of phenotypic traits that have been associated with airborne transmission of A/H5N1 virus, such as receptor binding preference, HA thermostability and acid stability, and polymerase activity was performed. *In vivo*, the potential of A/H5N6 BHG to transmit via the airborne (respiratory droplets and droplet nuclei) route between ferrets was assessed, as well as its pathogenicity in the ferret model. A/H5N6 BHG was found to not be transmissible via the airborne route but showed a remarkable tropism for extra-respiratory organs and was lethal in the ferret model.

## RESULTS

### A/H5N6 BHG causes severe disease in the ferret model

To assess the pathogenicity of A/H5N6 BHG, six adult female ferrets were inoculated intranasally with 10^6^ median tissue culture infectious dose (TCID_50_) of virus. Body weight and clinical signs were recorded daily. Nose and throat swabs were collected daily. Three animals were scheduled to be sacrificed and necropsied at day 3 and 6 days post-inoculation (dpi) each; however, one animal that was scheduled to be necropsied at 6 dpi had to be euthanized at 5 dpi because of a predefined humane endpoint of this experiment (20% weight loss compared to day 0). To be able to compare lesions and virus distribution at 3 and 6 dpi with an equal number of animals, the donor animal from the transmission experiment (see below, marked with an asterisk in Fig. 3) that died at 6 dpi was included in these analyses.

**FIG 1:**
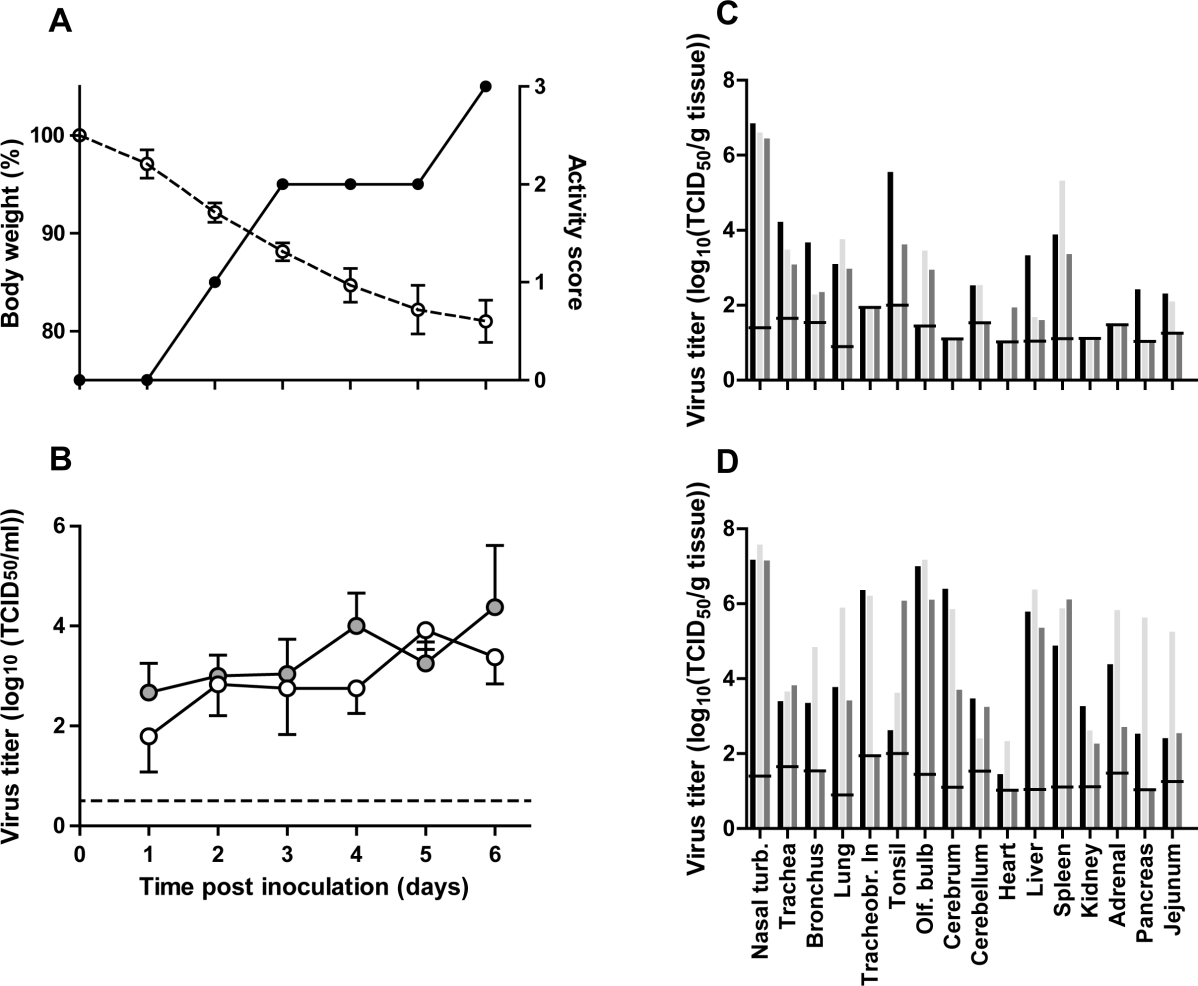
Average clinical scores, body weight, and virus titers in ferrets upon intranasal inoculation with A/H5N6-BHG. (**A**) Activity status and body weight. Activity status was scored daily as follows: 0, alert and playful; 1, alert and playful only when stimulated; 2, alert but not playful when stimulated; 3, neither alert nor playful when stimulated. (**B**) Virus titers in the throat (gray circles) and nasal swabs (white circles). (**C**) Virus titers in tissues of individual ferrets at 3 dpi. (**D**) Virus titers in tissues of individual ferrets at 6 dpi. All titers were determined by endpoint titration in Madin-Darby canine kidney cells. The lower limit of detection is indicated by the lines. All error bars represent standard deviation. Nasal turb., nasal turbinates; Tracheobr. ln, tracheobronchial lymph node; Olf. bulb, olfactory bulb.

A/H5N6 BHG-inoculated animals had lost on average 12% of their starting bodyweight at 3 dpi. The two remaining animals at 6 dpi had lost 19% and 20% of their starting body weight (Fig. 1A). At 3 dpi, all animals were alert but not playful when stimulated (Fig. 1A). From 4 dpi onward, all ferrets were presented with ruffled fur and with weak hind legs, and they kept their eyes slightly closed. At 6 dpi, the two remaining animals were lethargic and were breathing with exaggerated abdominal movement. The movement (walking) of one animal was severely deviating from routine because of a decrease in strength, while the other animal was not responsive.

Virus titers in nose and throat swabs were determined by endpoint titration in Madin-Darby canine kidney (MDCK) cells. Infectious virus shedding was demonstrated from 1 dpi onward and continued with a small increase in titer until 6 dpi in both samples (Fig. 1B). A/H5N6 BHG was detected by virus titration at high titers in the nasal turbinates, both at 3 and 6 dpi (mean titers of 10^6,6^ and 10^7,3^ TCID_50_/g of tissue, respectively) (Fig. 1C and D). Moreover, infectious A/H5N6 BHG was detected at 3 dpi in more tissues of the respiratory tract, such as the trachea (3/3), bronchus (3/3), lung (3/3), and tonsils (2/3), but also some extra-respiratory tract tissues, such as the liver and spleen in three out of three animals, the olfactory bulb, cerebellum, and jejunum in two out of three animals, and the heart and pancreas in one out of three animals. At 6 dpi, the A/H5N6 BHG infection had spread more widely into extra-respiratory tract tissues with all collected (extra-) respiratory tract tissues being virus-positive in titrations in at least two out of three animals. The virus dissemination in the ferret that was euthanized at 5 dpi was, based on titration data from the tissues collected from the ferrets, in between that of the 3 and 6 dpi animals.

### A/H5N6 BHG antigen-positive cells were present in respiratory, nervous, and lymphoid tissues

**FIG 2:**
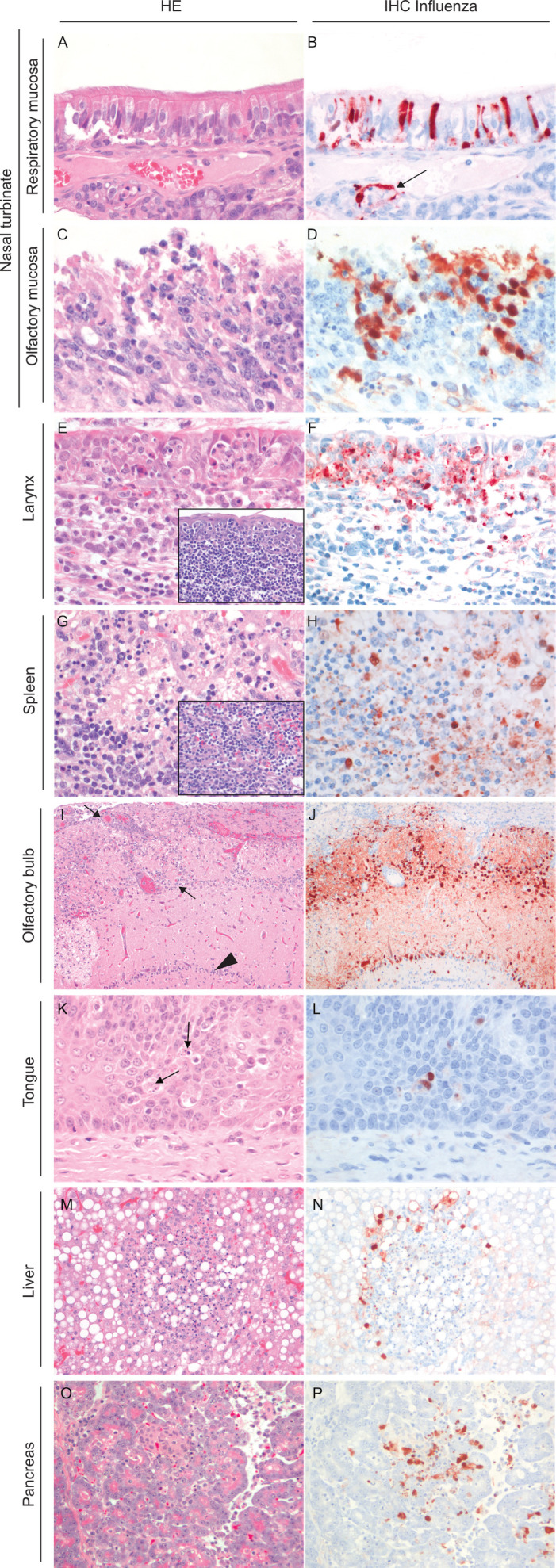
Lesions and virus antigen expression in several tissues inside and outside the respiratory tract of ferrets inoculated with A/H5N6 BHG/NL/17. Serial sections of tissues were stained with HE (left) or immunohistochemistry (right) with a monoclonal antibody against nucleoprotein of influenza A virus as primary antibody, visible as red staining. Magnification, ×400 (A–H, K–P) or ×100 (I and J). (A–D) Nasal turbinates. While olfactory mucosa has marked virus-associated damage, the mucosa covered by respiratory epithelium does not. Arrow indicates antigen positive submucosal gland. (E–H) Larynx mucosa overlying larynx lymphoid tissue and spleen. Insets show normal tissue architecture of uninfected ferret as reference. Larynx mucosal epithelial cells overlying the lymphoid tissue abundantly express virus antigen. Virus antigen expression in larynx and spleen is associated with moderate necrosis, lymphoid tissue is replaced by empty space and fibrin. (I and J) Olfactory bulb. Antigen expression is abundant in the neurons directly connected with olfactory epithelium, the periglomerular cells (cells in area between arrows), and the mitral cells (row of cells indicated with arrowhead). (K and L) Tongue. Antigen expression in tongue epithelium in area with taste buds suggests it can use neuroepithelial cells of the taste buds, like neuroepithelial cells of the olfactory mucosa, as a route for spread to the brain in ferrets. Antigen-positive cells co-localize with necrotic cells (arrows). Magnification ×400. (M–P) Liver and pancreas. Well-circumscribed foci of necrosis are associated with virus antigen within and bordering the lesion.

The upper respiratory tract was the body site that most consistently expressed abundant virus antigen. Virus antigen was detected in the upper respiratory tract of all ferrets at 3, 5, and 6 dpi. Within the upper respiratory tract, virus antigen was located in the nasal turbinates and the larynx in all ferrets. The cells that expressed virus antigen in the nasal turbinates were mainly clusters of olfactory and respiratory epithelial cells, and few individual submucosal gland epithelial cells and submucosal mononuclear cells (Fig. 2A through D). The virus antigen-positive cells in the larynx were epithelial cells overlying submucosal lymphoid tissue (Fig. 2E and F).

The lower respiratory tract inconsistently expressed virus antigen on day 3. Virus antigen was only present in 2/3 ferrets at 3 dpi, and in just one ferret at 6 dpi. The cells that expressed virus antigen in the lower respiratory tract were few clusters of adjacent bronchial and bronchiolar epithelial cells with occasional bronchial gland epithelial cells, also individual type I and type II pneumocytes and few individual alveolar macrophages expressed virus antigen (Table 1). These clusters of antigen-positive cells seemed randomly distributed.

**TABLE 1:** Distribution of influenza A virus antigen in tissues of ferrets inoculated intranasally with A/H5N6 BHG virus

Tissue^*a*^	Day 3 (*n* = 3)	Day 5 (*n* = 1)	Day 6 (*n* = 3)
Upper respiratory tract			
Nose			
Stratified epithelium	−^*b*^	±	−
Respiratory epithelium	+ (3/3)^*c*^	+^*c*^	+ (3/3)^*c*^
Submucosal glands (respiratory)	± (1/1)^*c*^	+^*c*^	+ (1/2)^*c*^
Olfactory epithelium	+ (3/3)^*c*^	+^*c*^	+ (3/3)^*c*^
Submucosal or Bowman’s glands (olfactory)	+ (3/3)^*c*^	+^*c*^	+ (3/3)^*c*^
Larynx			
Respiratory epithelium	+ (3/3)^*c*^	+^*c*^	+ (3/3)^*c*^
Lower respiratory tract			
Trachea	−	−	−
Bronchus	+ (1/3)^*c*^	−	± (1/3)
Bronchioles	+ (2/3)^*c*^	−	−
Alveoli	+ (2/3)^*c*^	−	± (1/3)
Nervous system			
Olfactory bulb			
Mitral cell (second-order neuron, sensation olfaction)	−	+^*c*^	+ (3/3)^*c*^
Periglomerular cell (second-order neuron, sensation olfaction)	± (2/3)^*c*^	+^*c*^	+ (3/3)^*c*^
Granule cell (third-order neuron, sensation olfaction)	−	+^*c*^	+ (3/3)^*c*^
Trigeminal ganglion (first-order neuron, sensation head)^*d*^	± (1/3)^*c*^	+^*d*^	± (3/3)^*c*^
Brainstem^*e*^			
Mesencephalic trigeminal nucleus (second-order neuron, sensation jaw)	−	n.a.^*i*^	+ (2/2)^*c*^
Nucleus caeruleus	−	n.a.	+ (1/2)^*c*^
Nucleus of the superior olive (second-order neuron, sensation from auditory system)	−	n.a.	+ (2/2)^*c*^
Vestibular nucleus (second-order neuron, sensation from auditory system)	−	n.a.	+ (1/1)^*c*^
Solitary nucleus (second-order neuron, sensation taste)	−	+^*c*^	+ (1/2)^*c*^
Parabrachial nucleus (third-order neuron, sensation taste)	−	+^*c*^	+ (1/1)^*c*^
Reticular formation	−	n.a.	+ (1/1)^*c*^
Enteric nervous system	−	−	± (2/3)^*c*^
Lymphoid system			
Spleen	± (2/3)^*c*^	±^*d*^	+ (3/3)^*c*^
Respiratory- or enteric-associated peripheral lymph node^*f*^	+ (1/2)^*c*^	n.a.	+ (2/2)^*c*^
Laryngeal-associated lymphoid tissue	+ (3/3)^*c*^	±^*c*^	± (3/3)
Gut-associated lymphoid tissue	−	±	+ (2/3)^*c*^
Bone marrow	−	n.a.	+ (2/3)^*c*^
Alveolar macrophages	+ (2/3)	−	−
Other tissues			
Liver	± (3/3)^*c*^	±^*c*^	+ (3/3)^*c*^
Pancreas^*g*^	−	±^*c*^	+ (1/2)^*c*^
Adrenal gland^*h*^	+ (1/2)^*c*^	−	−

^*a*^ Virus antigen expression was not observed in heart, kidney, esophagus, or epithelial cells of duodenum, jejunum, and colon of any of the ferrets.

^*b*^ −, virus antigen was not detected; ± <10 cells contained virus antigen; +, >10 cells contained virus antigen.

^*c*^ The detection of virus antigen was associated with histological lesions.

^*d*^ The trigeminal ganglion of one ferret was not sampled.

^*e*^ Locations of specific nuclei were based on topography and morphology. The sagittal level of the brain section differed for the ferrets. Therefore, comparison was not always possible for all regions.

^*f*^ No peripheral lymph node was sampled for one ferret at 3 , 5, and 6 dpi.

^*g*^ No pancreas was sampled for one ferret at 6 dpi.

^*h*^ No adrenal gland was sampled for one ferret at 3 and 5 dpi.

^*i*^ n.a., not applicable

The lymphoid (Fig. 2E through H) and nervous tissues (Fig. 2I and J) were the second most common to be abundantly antigen positive. In the nervous tissue, virus antigen was most consistently present in olfactory bulbs and less consistently in the trigeminal ganglion, brainstem, and enteric plexi. The increased proportion of antigen-positive cells in olfactory bulb and trigeminal ganglion at 5 and 6 dpi compared to 3 dpi suggests an increased infection of both these sites over time. Areas expressing virus antigen in the brainstem and enteric plexi were only positive later in the course of the experiment, at 5 and 6 d pi, and not in any of the ferrets at 3 dpi. The cells that expressed antigen in the nervous tissues were interpreted based on morphology and their locality related to other cells as being both neurons and glial cells. In one ferret at 6 dpi, a small focus of ependymal cells expressed virus antigen. Aside from these ependymal cells, virus antigen was absent in cerebrum and cerebellum of all brain sections evaluated.

In the nervous tissue, virus antigen was present in locations suggesting virus had spread there via neuronal connections: virus was most consistently present in the olfactory bulbs, which are directly connected with the olfactory epithelium in the nose. Virus was also present in the trigeminal ganglion, which contains the cell bodies of the sensory neurons that innervate the head. Furthermore, at 5 and 6 dpi, several distinct, well-demarcated foci of antigen-positive cells were present in the brainstem. The sagittal plane of the brain section differed for these four ferrets, and therefore comparison was not always possible for all regions. If a brain region was positive in one ferret, and this brain region was also present in the brain slide of another ferret, it was mostly also positive in that other ferret. This strongly suggests that the distribution was not random and that virus did not disseminate to the brain via the circulation. Aside from the reticular formation, all mentioned nuclei contain second- or third-order neurons from sensory cranial neurons innervating the nose, mouth, or ear, suggesting this influenza virus did not only spread via the olfactory epithelium to the brain but also via other sensory neuron routes. To examine the possibility that virus might have disseminated via neurons from the taste buds on the tongue to the brainstem, retrospective examination of taste buds in the vallate papillae of tongue tissues was performed. Vallate papillae (tongue) were sampled in formalin for five of the seven ferrets. Antigen-positive cells were detected in the epithelial layer in an area with taste buds and co-localized with necrotic cells and neutrophils in one ferret (Fig. 2K and L). Furthermore, taste buds contained necrotic cells and neutrophils in three of the other four ferrets. This suggests that indeed sensory neurons like those involved in taste sensation were involved in the spread of influenza virus to the brain.

In the lymphoid tissue, virus antigen was detected in all ferrets. The cells that expressed virus antigen in lymphoid tissues were individual or groups of variably shaped mononuclear cells with large nuclei and abundant cytoplasm. Based on morphology, these cells were likely macrophages or dendritic cells (Table 1; Fig. 2G and H). Adrenal gland, liver, and pancreas also contained virus-antigen-positive cells, which were identified as parenchymatous cells (Fig. 2M through P).

### Ferrets inoculated with A/H5N6 BHG had marked histological lesions associated with virus infection

Association of histological lesions and A/H5N6 BHG infection was based on co-localization of lesions and virus-antigen expression. Lesions associated with A/H5N6 BHG infection generally consisted of a central area of advanced necrosis (characterized by loss of tissue architecture, increased intercellular spaces, karyorrhectic debris, and eosinophilic material), bordered by an area where fewer cells showed necrosis and the presence of small numbers of neutrophils. This outer area generally had virus-antigen-positive cells.

Lesions associated with virus antigen in the upper respiratory tract consisted of a mild to moderate multifocal necrotizing rhinitis (Fig. 2A through D) and laryngitis (Fig. 2E and F), characterized at 3 dpi by necrosis of olfactory, respiratory, and glandular epithelial cells, and infiltration of mild to moderate numbers of neutrophils. Exudate composed of cell debris and mucus covered the surface. At 5 and 6 dpi, lesions were similar as at 3 dpi but extended deeper into the submucosa.

In the lower respiratory tract, lesions associated with virus antigen were mild and multifocal and were centered around bronchioles. Bronchioles and alveoli showed necrosis and infiltration of neutrophils, and there was an increase in the number of intra-alveolar macrophages in affected areas. The ferrets that were euthanized at 5 and 6 dpi showed no lung lesions, suggesting that lower respiratory tract infection had not been more severe at 3 dpi than scored at time of euthanasia in these two ferrets (Table 1).

Lesions associated with virus antigen in the nervous tissues were present in the olfactory bulb (Fig. 2I and J), trigeminal ganglion, and brainstem and were generally very mild or absent in ferrets at 3 dpi, while mild to marked in ferrets at 5 and 6 dpi. Lesions consisted of foci of neuronal and glial necrosis, characterized by swelling and rounding of the cell bodies, hypereosinophilia, karyorrhexis, karyolysis, and increased intercellular spaces. Additionally, there was infiltration by small numbers of neutrophils and, if blood vessels were close to lesions, mild perivascular cuffing with lymphocytes. There was no lesion associated with the neuronal cells that were antigen-positive in the intestine.

Also associated with virus antigen were lesions in spleen- and larynx-associated lymphoid tissue, liver, pancreas, and adrenal gland. These lesions consisted of necrosis of parenchymal cells and infiltration with neutrophils (Fig. 2M through P). In the liver-, spleen-, and larynx-associated lymphoid tissues, lesions were mild or absent at 3 dpi and marked at 5 and 6 dpi. Pancreas and adrenal gland lesions were less consistent, and therefore an increase in severity of lesions over time could not be observed in these tissues.

Lesions indirectly associated with A/H5N6 BHG-positive cells were present in one ferret (6 dpi). This ferret had a lesion in the gut-associated lymphoid tissue (GALT) of the jejunum that was most likely related to A/H5N6 BHG infection because the character of the lesion fitted with the lesions in other lymphoid tissues, where antigen-positive cells were present. The overlying mucosa of the jejunum was ulcerated allowing the necrotic GALT to have direct contact with the intestinal lumen. Coccoid bacteria were present in between necrotic cells in the GALT. Within the same section of jejunum, bacteria, both rods and cocci, were present in blood vessels, suggesting bacteremia had occurred prior to euthanasia.

Other histological lesions not associated with A/H5N6 BHG infection were present in liver, kidney, adrenal gland, skin, intestine, and spleen. All ferrets had a chronic mild (1/3 ferrets at 3 dpi, 3/3 ferrets at 6 dpi), moderate (1/3 ferrets at 3 dpi, 1/1 ferret at 5 dpi), or marked (1/3 ferrets at 3 dpi) lymphoplasmacytic portal hepatitis. All ferrets had hepatic lipidosis. One ferret (3 dpi) had chronic moderate multifocal lymphoplasmacytic perivascular infiltrates in the adrenal gland. One ferret (3 dpi) had mild multifocal mineralization in the interstitium of the kidney. One ferret (5 dpi) had a multifocal superficial necropurulent dermatitis. Two ferrets (6 dpi) showed villus blunting and fusion in duodenum and jejunum. All ferrets had mild to moderate extramedullary hematopoiesis in the spleen, characterized by the presence of megakaryocytes in the spleen.

### Amino acid substitution D701N in PB2 was positively selected in all ferrets

The high lethality and extensive tissue tropism of A/H5N6 BHG in ferrets prompted us to investigate whether any amino acid changes had occurred in the virus as the result of ferret adaptation or the positive selection of minority variants in the virus inoculum. From all six ferrets in the pathogenesis experiment that were euthanized at 3, 5, or 6 dpi, viral RNA was extracted from nasal turbinates, lung, cerebrum, and spleen, followed by a multi-segment RT-PCR and next-generation sequencing on a MiSeq instrument (Illumina, Cambridge, UK) as described previously (25). The MDCK passage 2 virus stock that was used for inoculation was also sequenced. For the virus stock and the ferret samples, the threshold for mutation detection was set at 1% and 5%, respectively. Many silent and non-silent mutations were identified as compared to the consensus sequence of the original material (Table S1 and reference sequence EPI1131093-EPI1131100). Eight substitutions that were present as a minor variant (<5%) in the virus inoculum were found to be present in two or more ferrets with a frequency of >5% (Table 2). One of these substitutions was the well-known D701N substitution in PB2, a mammalian adaptation marker that has previously been described to increase H5N1 virus polymerase activity and replication in mammalian cells (26). Interestingly, D701N was only present as a minor variant in the virus inoculum (2.4%) but was positively selected in all investigated tissues (range 23.7%–99.4%, average 59.8%) except for one. Remarkably, in this cerebrum sample from ferret 4 (D701*N* <5%), two other substitutions emerged in PB2, which were exclusively detected in this sample, G590S and I647M (Table 2). It is possible that these substitutions have a compensatory effect for the absence of D701N. Furthermore, the K339E substitution that was only present in 1.3% of the sequences in the inoculum was present in 17 out of the 22 sequenced ferret samples at a frequency higher than 5% (range 7.2%–82.2%, average 32.4%), which suggests that this substitution was also beneficial for efficient replication in ferrets.

**TABLE 2:** Deep sequencing analysis of A/H5N6 BHG inoculum and ferret tissues^*a*^

		Percentage of reads having the indicated substitution									
Gene segment		PB2							PB1		NA
AA substitution		K80R	E188G	K339E	S489P	G590S	I647M	D701N	P596S	P708S	E268K
	MDCK2 stock	<1^*b*^	3.2	1.3	<1	<1	<1	2.4	2.3	<1	<1
Ferret 1 (3 dpi)	Nasal turb.	<5^*c*^	5.5	7.2	<5	<5	<5	23.7	<5	<5	<5
	Lung	17.1	<5	41.9	5.9	<5	<5	35.8	<5	12.3	<5
	Cerebrum	10.9	<5	<5	<5	<5	<5	35.8	<5	<5	<5
	Spleen	<5	<5	<5	<5	<5	<5	87.2	<5	<5	<5
Ferret 2 (3 dpi)	Nasal turb.	<5	<5	10.0	<5	<5	<5	29.5	<5	<5	<5
	Lung	<5	<5	<5	<5	<5	<5	43.7	10.9	<5	<5
	Cerebrum	ND^*d*^	ND	ND	ND	ND	ND	ND	ND	ND	ND
	Spleen	<5	<5	82.2	<5	<5	<5	52.9	<5	<5	20.8
Ferret 3 (3 dpi)	Nasal turb.	<5	<5	10.7	<5	<5	<5	29.1	<5	<5	<5
	Lung	<5	43.0	13.8	<5	<5	<5	58.5	<5	<5	<5
	Cerebrum	<5	<5	50.5	<5	<5	<5	43.9	<5	<5	<5
	Spleen	<5	<5	19.4	<5	<5	<5	89.6	<5	<5	<5
Ferret 4 (5 dpi)	Nasal turb.	ND	ND	ND	ND	ND	ND	ND	ND	ND	ND
	Lung	<5	<5	41.9	<5	<5	<5	94.7	<5	<5	<5
	Cerebrum	<5	<5	<5	<5	92.2	92.9	<5	<5	<5	<5
	Spleen	<5	<5	11.9	<5	<5	<5	97.5	<5	<5	<5
Ferret 5 (6 dpi)	Nasal turb.	<5	<5	11.9	<5	<5	<5	37.6	<5	<5	<5
	Lung	<5	<5	56.8	<5	<5	<5	99.1	<5	<5	<5
	Cerebrum	<5	<5	<5	<5	<5	<5	99.4	<5	<5	<5
	Spleen	<5	<5	63.3	<5	<5	<5	98.5	<5	<5	6.2
Ferret 6 (6 dpi)	Nasal turb.	<5	<5	17.7	<5	<5	<5	49.8	<5	<5	<5
	Lung	<5	<5	44.5	<5	<5	<5	26.1	<5	<5	<5
	Cerebrum	24.8	<5	19.9	22.7	<5	<5	38.7	26.5	9.8	<5
	Spleen	<5	6.0	47.4	<5	<5	<5	85.4	<5	<5	<5

^*a*^ Substitutions that were <5% present in the virus genome of the MDCK2 virus stock, but >5% present in two or more ferret samples are shown. In addition, two substitutions are depicted that were present in the cerebrum of ferret 4, the only sample that did not contain the D701N substitution. Nasal turb., nasal turbinates.

^*b*^ The detection limit of the MDCK2 virus stock was set at 1%.

^*c*^ The detection limit of the ferret tissues samples was set at 5%.

^*d*^ Not done, no PCR products were amplified from this material.

### A/H5N6 BHG does not transmit between ferrets via the airborne route

**FIG 3:**
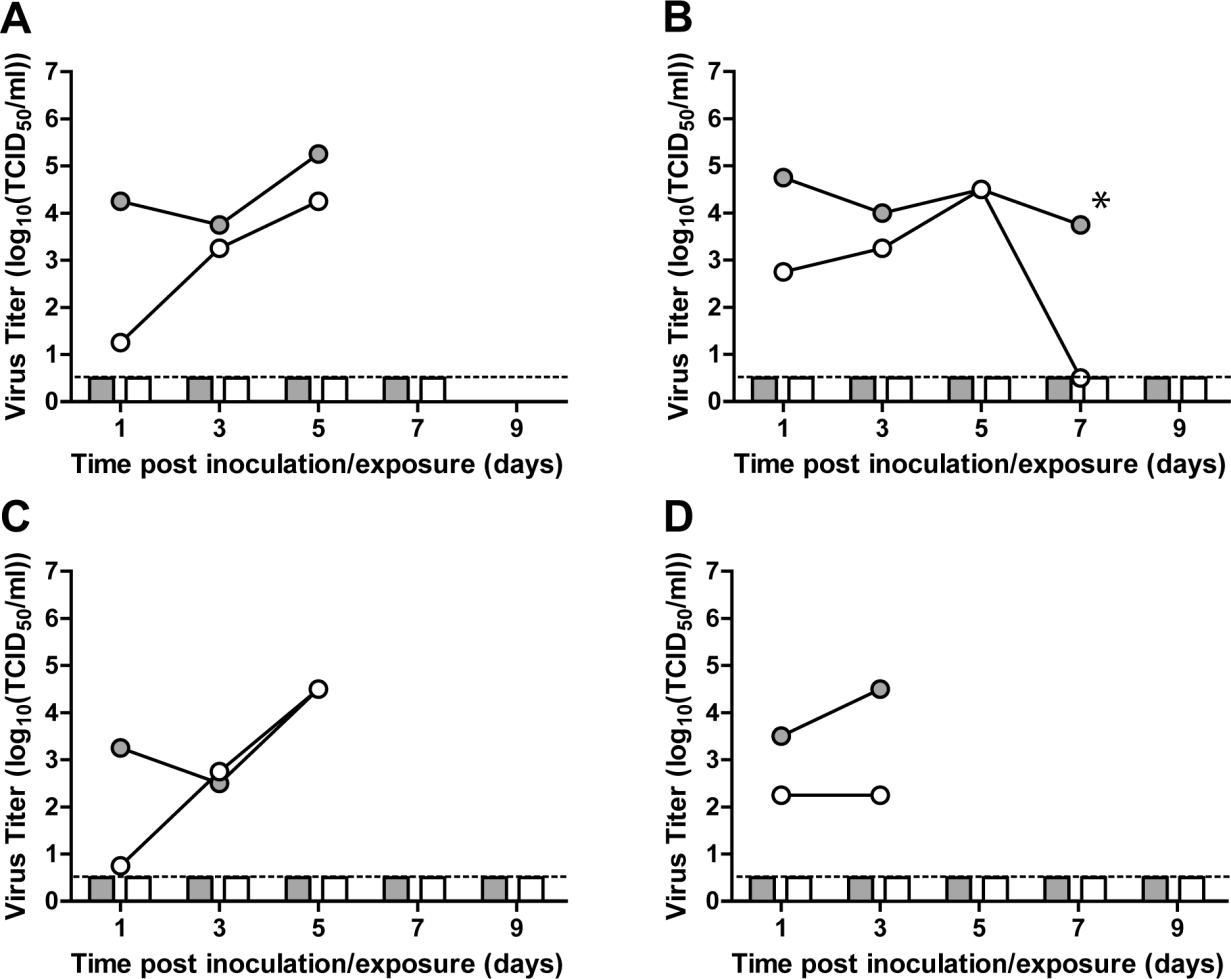
Lack of airborne transmission of A/H5N6-BHG between ferrets. Data for individual transmission experiments are shown in each panel (**A–D**), with virus shedding from donor and recipient ferrets shown as lines and bars, respectively. Donor animals were euthanized at 5 dpi (panel A and C) or succumbed to disease at 6 dpi (panel B) or 5 dpi (panel D, no swab collected). *, swabs from the donor animal were collected at 6 dpi; *, data from this animal were also used in the analysis of the pathogenesis experiment. Gray circles and bars represent shedding from the throat; white circles and bars represent shedding from the nose. The lower limit of detection is indicated by the dashed lines.

Human influenza viruses are transmitted among humans via the airborne route by aerosols or respiratory droplets. For public health risk assessment, it is, hence, important to investigate the airborne transmissibility of emerging influenza A viruses. Here, airborne transmission experiments were performed in the ferret model. Four separately housed donor ferrets were inoculated with A/H5N6-BHG, and 1 day later, a naïve recipient ferret was placed in a cage adjacent to each donor cage. Donor and recipient ferrets were separated by two steel grids, 10 cm apart, allowing viruses to be transmitted only via the airborne route. Virus shedding from the donor ferrets was moderate, as measured through nasal and throat swabs, with titers up to 10^5,25^ TCID50/mL (Fig. 3). However, two donor animals had to be euthanized at 5 dpi because they had reached the humane endpoint. The two other animals succumbed to the infection at 5 (no swabs collected) and 6 dpi, highlighting the fast progression of this disease in ferrets. Upon exposure, no replicating virus was detected in any of the recipient ferrets. Unexpectedly, one recipient animal died at 8 days post-exposure. This was likely not related to A/H5N6 infection because this ferret tested negative for virus in swabs from days 1 to 8 post-exposure (dpe). Lack of transmission was confirmed by the absence of seroconversion 14 dpe in the three remaining recipient ferrets (Fig. 3).

### A/H5N6 BHG binds to avian-type receptors

The influenza A virus HA protein binds to host cells via sialylated receptors. Avian and human influenza viruses bind α2,3-linked sialic acids (α2,3-SA, avian-type receptors) and α2,6-linked sialic acids (α2,6-SA, human-type receptors), respectively. To assess the receptor specificity of A/H5N6-BHG, a resialylated turkey red blood cell (TRBC) assay was performed using recombinant A/Puerto Rico/8/1934 (H1N1) viruses harboring the HA protein of interest. Briefly, TRBCs were treated with sialidase to strip off SA and subsequently modified to contain either α2,3-SA or α2,6-SA, using specific sialytransferases. Both control viruses, harboring the avian A/H5N1 virus A/Indonesia/5/2005 (Indo/05) HA and the human A/H3N2 virus A/Netherlands/213/2003 (NL/03) HA, displayed the expected receptor attachment pattern by binding exclusively to α2,3-SA and α2,6-SA, respectively (Table 3). A/H5N6-BHG displayed a typical avian phenotype by binding exclusively to α2,3-SA, comparable to two other previously described clade 2.3.4.4 viruses, A/H5N6 A/Guangzhou/39715/2014 (GZ/14) and A/H5N8 A/chicken/Netherlands/EMC-3/2014 (ck/NL/14) (13, 18).

**TABLE 3:** Receptor specificity of the different viruses as determined by a modified turkey red blood cell (TRBC) hemagglutination assay^*a*^

Virus	Untreated	VCNA	α-2.3	α-2.6
A/H3N2 NL/03	48	0	0	32
A/H5N1 Indo/05	12	0	16	0
A/H5N6 BHG/NL/17	16	0	24	0
A/H5N8 Ck/NL/14	24	0	24	0
A/H5N6 GZ/14	48	0	48	0

^*a*^ A resialylated (TRBC) assay was performed to assess the receptor specificity of influenza virus HA proteins. TRBCs were treated with VCNA sialidase to strip off sialic acids and subsequently modified to contain either α2,3-SA or α2,6-SA, using specific sialytransferases. The values show the hemagglutination titer of the viruses with the respective TRBCs. VCNA, *Vibrio cholerae* neuraminidase.

### A/H5N6 BHG HA is acid and temperature unstable

**FIG 4:**
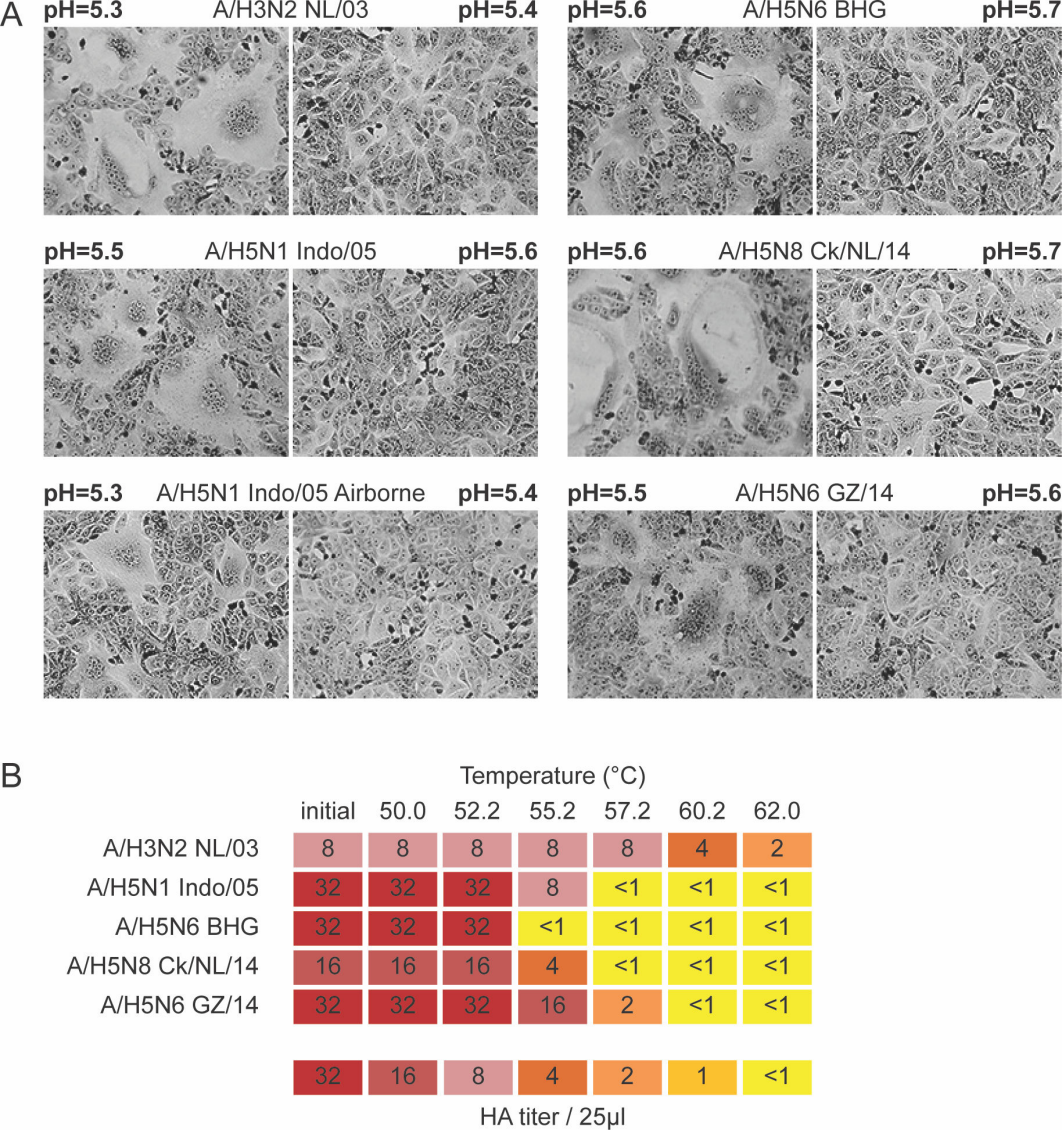
Acid stability and thermostability of clade 2.3.4.4 A/H5 and control A/H3 viruses. (**A**) Syncytium formation in Vero-118 cells upon expression of different HAs and acidification at different pH values. Pictures corresponding to the highest pH at which maximum syncytium formation was observed (defined as the pH threshold of fusion) and 0.1 pH unit higher are shown. (**B**) HA protein thermostability was measured by the ability of viruses to agglutinate TRBCs after incubation for 30 minutes at the indicated temperatures (Celsius). Colors indicate the HA titers upon treatment at various temperatures as shown in the color key (HA titer/25 µL).

Influenza virus attachment to SA receptors on the host cell surface is followed by receptor-mediated endocytosis. In endosomes, a low-pH-triggered conformational change of HA mediates fusion of the viral and endosomal membranes to release the viral genome in the cytoplasm (27). In order to assess the acid stability of the A/H5N6-BHG HA, a syncytium formation assay was performed to measure the pH threshold required for HA-mediated cell-to-cell fusion. Vero cells were transfected with plasmids expressing different HAs. The next day, the HA-expressing cells were exposed to trypsin to cleave and activate the HA, followed by acidification of the cell culture by a pH gradient ranging from pH 4.8 to 5.9. One day later, cell cultures were inspected visually for the presence of syncytia (multinucleated cells) to determine the pH threshold triggering the conformational change and subsequent membrane fusion (Fig. 4A). The A/H5N1 Indo/05 HA (unstable, pH 5.5), the A/H5N1 Indo/05 HA carrying airborne-transmission substitutions (H103Y/T156A/Q222L/G224S, H5 numbering throughout the manuscript) (stable, pH 5.3), and the human A/H3N2 NL/03 HA (stable, pH 5.3) were included as controls (28). The highest pH at which cell-to-cell fusion was triggered by A/H5N6-BHG was pH 5.6. A similar threshold pH for fusion was found for A/H5N6 GZ/14 HA (pH 5.5) and A/H5N8 ck/NL/14 HA (pH 5.6).

The irreversible conformational change of HA from a metastable non-fusogenic to a stable fusogenic state can also be triggered at neutral pH when the HA is exposed to increasing temperatures (29). Therefore, the thermostability was assessed using a temperature sensitivity assay to further assess HA stability (Fig. 4B). Viruses were exposed to a temperature gradient ranging from 50°C to 62°C for 30 minutes, and the HA titer was subsequently recorded. The stable post-fusion conformation of HA does not allow binding to receptors anymore, resulting in a negative HA-titer. In agreement with the results of the fusion assay, all avian-origin A/H5 HAs (A/H5N6-BHG, A/H5N1 Indo/05, A/H5N6 GZ/14, and A/H5N8 ck/NL/14) were less stable than the human A/H3N2 NL/03 HA.

### D701N substitution in PB2 increases the polymerase activity of A/H5N6 BHG

**FIG 5:**
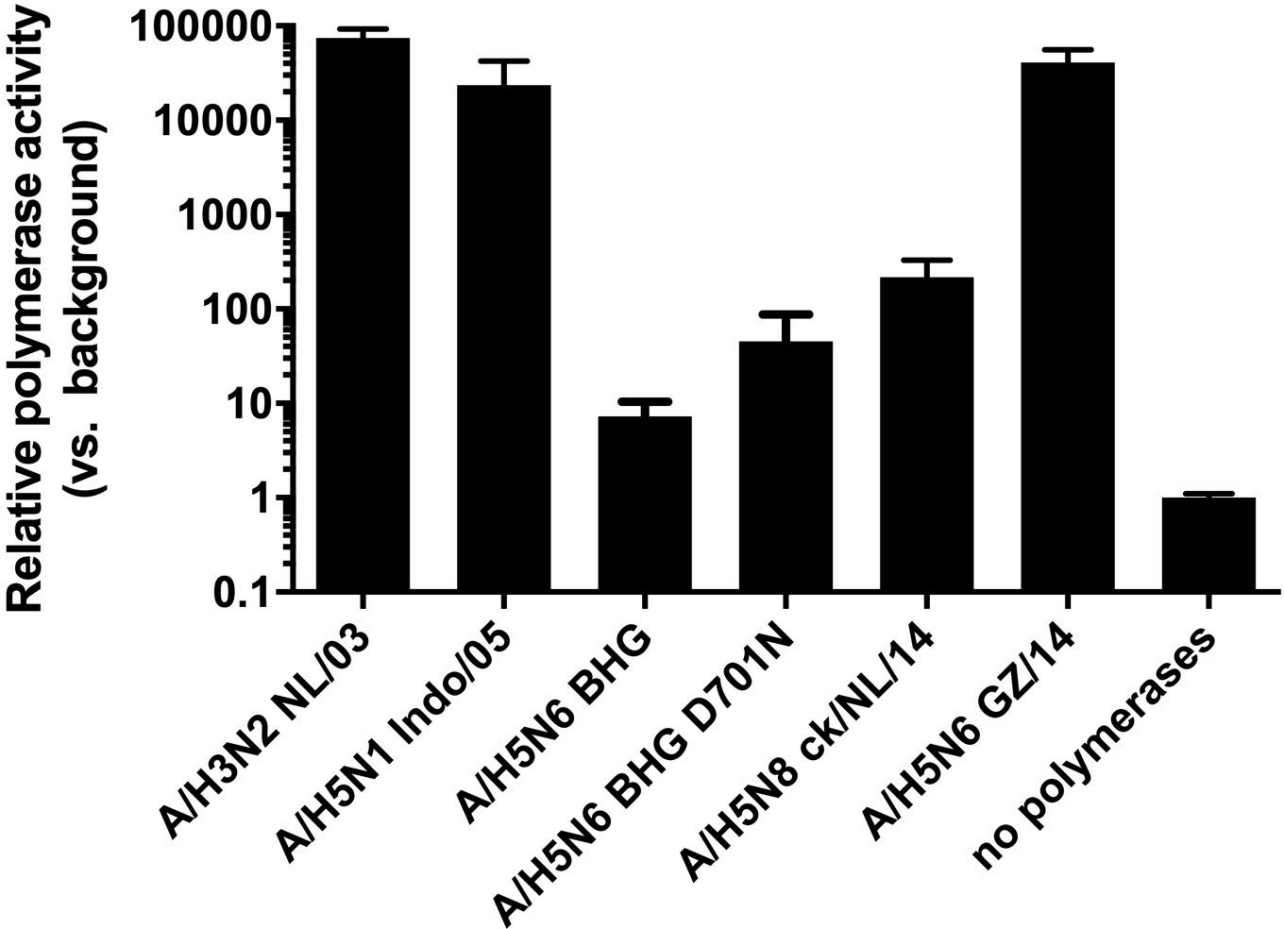
Polymerase activity of clade 2.3.4.4 A/H5 and control A/H3 viruses. 293T cells were transfected with plasmids containing PB2, PB1, PA, and NP genes, a firefly luciferase reporter plasmid, and a control *Renilla* luciferase plasmid. Each transfection was performed in quadruplicate in at least two independent experiments. After transfection, the 293T cells were cultured at 37°C for 24 h, and luciferase activity was measured in the cell extracts. Results are presented as the means ± standard deviations (error bars) of polymerase activity of virus compared to the negative control.

To measure the polymerase activity of A/H5N6 BHG, minireplicon assays were performed by transfecting 293T cells with plasmids expressing the polymerase genes (PB2, PB1, and PA) and the nucleoprotein (NP) of different A/H5 viruses, firefly (FF) luciferase as a reporter gene and *Renilla* luciferase as an internal control. The experiment was performed at 37°C. Polymerase activities were expressed as fold increase compared to the negative control. The polymerase activity of the A/H5N6-BHG was only approximately sevenfold higher than the control, which is much lower than the human and avian control polymerase complex activities (approximately 10,000–100,000-fold increase) (Fig. 5). Introduction of the D701N substitution in the A/H5N6-BHG PB2 that was positively selected in all ferrets resulted in a sixfold increase in polymerase activity as compared to the wild-type (WT) PB2 (45-fold increase compared to the control). This indicates that the D701N substitution is beneficial for the polymerase activity of A/H5N6-BHG; however, this activity was still lower than that of the A/H5N8 ck/NL/14 and A/H5N6 GZ/14 (5-fold and 900-fold higher than A/H5N6-BHG D701N). It should be noted that A/H5N6 GZ/14 possesses a lysine at position 627 in PB2, which has been associated with adaptation of avian viruses to mammalian hosts (30 - 32). Nevertheless, it was previously described that the avian genotype PB2 K627E had a 32 times lower polymerase activity than that of WT A/H5N6 GZ/14, which is still much higher than that of A/H5N6-BHG D701N (18).

## DISCUSSION

Here, we report a detailed *in vitro* and *in vivo* characterization of a clade 2.3.4.4 A/H5N6 virus, that was isolated from a black-headed gull in the Netherlands in 2017 (A/H5N6-BHG), to assess the potential risk for humans. We first assessed the pathogenicity and transmissibility of A/H5N6-BHG in the ferret model. Ferrets generally show similar susceptibility to infection with human and avian influenza viruses and develop respiratory disease similar to that observed in humans (33 - 35). Airborne transmission (i.e., via respiratory droplets and droplet nuclei) is a main route for influenza virus transmission between humans. For this reason, it is crucial to investigate the airborne transmissibility of emerging influenza A viruses. A/H5N6-BHG replicated relatively well in the donor ferrets, with peak titers in swabs up to 10^5.25^ TCID50/mL (Fig. 3). However, as a result of the high pathogenicity of the virus, two donor animals had to be euthanized at 5 dpi, whereas the other two animals died at 5 and 6 dpi. A/H5N6-BHG was not transmitted via the airborne route as demonstrated by the absence of replicating virus (Fig. 3) and absence of seroconversion (HI titers <10) in the three recipient ferrets. This is in accordance with other studies on transmissibility of genetically distinct A/H5N6 viruses, which reported direct contact transmission in four out of five ferret studies but lack of airborne transmission in five ferret studies (18 - 23).

Intranasal inoculation of ferrets with A/H5N6-BHG resulted in severe disease and mortality, with 20% weight loss in 5–6 days in some ferrets (Fig. 1A). In previous studies on the pathogenicity of A/H5N6 viruses, disease severity was strongly dependent on the viral strain used, ranging from no or relatively mild clinical signs, such as coughing and sneezing (17, 19, 21, 22), inactivity or ruffled fur but with regained activities and stabilized body weight at 6 dpi (18) to severe disease accompanied with 13%–14% body weight loss with diarrhea and neurological complications requiring humane endpoint (23). In the present study, at 6 dpi, infectious virus was detected in almost all tissues collected for each ferret, and virus detection was usually associated with marked histopathologic changes. In other studies on the pathogenicity of avian A/H5N6 viruses, extra-respiratory spread was highly dependent on the virus strain used. Virus was detected in the spleen of ferrets in two out of four studies performed (17, 19, 21, 22). Kwon et al. also detected virus in the brain but not in the kidney, heart, and colon (19). Noh et al. collected extra-respiratory tissues such as intestine, kidney, and spleen, but no virus was detected (21). Some of the ferrets inoculated with a human A/H5N6 virus were positive in extra-respiratory tissues, such as olfactory bulb, cerebrum, cerebellum, pancreas, and jejunum but at very low titers (18). In contrast, A/H5N6 A/Sichuan/26221/2014 and A/duck/Bangladesh/19D770/2017 viruses were detected in the spleens, livers, intestines, olfactory bulbs, and brains of some animals reaching humane endpoints (23).

The consistent detection of lesions associated to virus antigen in lymphoid tissues in our experiments (Table 1, Fig. 2) was unexpected. Virus antigen in lymphoid tissues has been detected previously in mammals experimentally infected with H5N1, these included cats (36), foxes (37), macaques (38), and ferrets (39). However, in all these animals, microscopic lesions associated to the antigen were not detected (36, 37) or were inconsistently present (38, 39). While in mammals highly pathogenic avian influenza viruses thus do not seem to cause lesions in lymphoid tissues readily, in birds they do (40, 41). If necrosis in the lymphoid tissues is severe enough, it will have a major effect on the course of the infection. Necrosis of lymphocytes and macrophages can be expected to hamper the development of an adaptive immune response, which is necessary to fight off the influenza virus infection. Therefore, the finding that A/H5N6 BHG consistently causes lesions in all lymphoid tissues that were examined in our ferrets, is reason for concern and should be further studied.

To investigate the basis of the unexpected high pathogenicity and systemic replication in ferrets, A/H5N6-BHG was further characterized *in vitro* to search for potential phenotypic differences compared to other A/H5 viruses. Three phenotypic properties that have previously been associated with pathogenicity, mammalian adaptation, and airborne transmission of A/H5N1 were studied: HA receptor binding preference, HA thermostability and acid stability, and polymerase activity (28, 42).

An important characteristic of adaptation of avian influenza viruses to mammalian species is to gain binding to human-type α2,6-SA receptors. Most clade 2.3.4.4 viruses were previously shown to have α2,3-SA specificity. Using a resialylated TRBC assay, A/H5N6-BHG was shown to also display a typical pattern of avian influenza viruses by binding exclusively to α2,3-SA. These data are in agreement with previous studies, in which an avian and a human A/H5N6 isolate were found to bind only to α2,3-SA (43, 44). Interestingly, dual-receptor-binding specificity has been demonstrated for A/H5N6 viruses isolated from ducks and chickens in China, and A/H5N6 viruses isolated from wild bird carcasses in Hong Kong (20, 22, 43).

A second important phenotype that is associated with adaptation of avian influenza viruses to humans and pandemic potential is HA acid stability (28, 42, 45). The A/H5N6-BHG HA did not contain any of the previously described substitutions that are known to increase the acid stability and thermostability of A/H5 HAs, such as H103Y, T315I, and K58I (28, 42, 45). It also did not contain the deletion at position 131, often observed in HAs from human H5N6 virus and recently described to stabilize the HA trimer and pre-fusion state by introducing an N-linked glycosylation (46). The HA of A/H5N6-BHG was unstable compared to that of a human A/H3N2 virus and had a similar stability as three other A/H5 HAs tested, as assessed in syncytium formation and thermostability assays (Fig. 4).

A high polymerase activity in human cells is also important for adaptation of avian influenza A viruses to the new host. A/H5N6-BHG possessed a low polymerase activity as compared to other A/H5 viruses tested, and even the PB2 D701N substitution that was positively selected in all ferrets did not increase the polymerase activity to the activity of the other A/H5 viruses tested in minireplicon assays (Fig. 5).

Collectively, our results demonstrated that the Dutch HPAI H5N6 A/black-headed gull/Netherlands/29/2017 virus replicated systemically in ferrets with an unusual lymphotropism and neurotropism, had high pathogenicity but lacked the ability to transmit via the airborne route in the ferret model. The high pathogenicity could not be explained by our phenotypical *in vitro* analyses and warrants further investigation.

Careful extrapolation from animal models to humans suggests that the public health threat of the HPAI H5N6 viruses is low. However, given the rapid geographical spread of HPAI clade 2.3.4.4 A/H5Nx viruses, including recent clade 2.3.4.4b A/H5N1 viruses, their ability to infect various species of birds and mammals including humans, and the tendency of influenza A viruses to mutate and reassort, the phenotypic properties of HPAI H5Nx viruses should be monitored closely (47) (https://cdn.who.int/media/docs/default-source/influenza/avian-and-other-zoonotic-influenza/h5-risk-assessment-dec-2022.pdf?sfvrsn=a496333a_1&download=true).

## MATERIALS AND METHODS

### Cells

MDCK cells (ATCC) were cultured in Eagle’s minimal essential medium (EMEM, Lonza Benelux BV, Breda, the Netherlands) supplemented with 10% fetal bovine serum (Greiner Bio-One B.V., Alphen aan den Rijn, The Netherlands.), 100 U/mL penicillin (P, Lonza), 100 U/mL streptomycin (S, Lonza), 2 mM L-glutamine (L-glu, Lonza), 1.5 mg/mL sodium bicarbonate (NaHCO_3_, Lonza), 10 mM HEPES (Lonza), and 1× non-essential amino acids (NEAA, Lonza).

### Viruses

A/black-headed gull/Netherlands/29/2017 virus (EPI_ISL_289714) was isolated from a combined oropharyngeal and cloacal swab. The virus was then propagated for two passages in MDCK cells. Clade 2.3.4.4 viruses A/H5N6 A/Guangzhou/39715/2014 (GZ/14) and A/H5N8 A/chicken/Netherlands/EMC-3/2014 (ck/NL/14) have been described previously (13, 18).

### Virus titration in MDCK cells

Virus titrations were performed as described previously (33). Briefly, MDCK cells were inoculated with 10-fold serial dilution of virus stocks, nose swabs, throat swabs, or homogenized tissue samples. Cells were washed with PBS 1 h after inoculation and cultured in infection media, consisting of EMEM supplemented with 100 U/mL P, 100 U/mL S, 2 mM L-glu, 1.5 mg/mL NaHC0_3_, 10 mM HEPES, 1× NEAA, and 20 µg/mL trypsin (Lonza). Three days after inoculation, supernatants of cell cultures were tested for agglutinating activity using turkey erythrocytes as an indicator of virus replication. Infectious virus titers were calculated from four replicates each of the homogenized tissue samples, nose swabs, and throat swabs and for 10 replicates of the virus stocks by the method of Spearman-Karber.

### Transmission experiment

Airborne transmission experiments were performed as described previously (33, 48). In short, four adult female ferrets were inoculated intranasally under anesthesia with 10^6^ TCID_50_ of virus by applying 250 µL of virus suspension to each nostril. Each donor ferret was then placed in a transmission cage. One day after inoculation, one naïve recipient ferret was placed opposite each donor ferret. Each transmission pair was housed in a separate transmission cage designed to prevent direct contact but allowing airflow from the donor to the recipient ferret. Nose and throat swabs were collected every other day from donor ferrets until the humane endpoint was reached and at 1, 3, 5, 7, and 9 dpe from the recipient ferrets and subjected to virus titration. Recipient ferrets were euthanized at 14 dpe to allow assessment of seroconversion.

### Pathogenesis in ferrets

Six adult female ferrets were inoculated intranasally with 10^6^ TCID_50_ of HPAI A/black-headed gull/Netherlands/29/2017. Clinical scores were assessed every day. Activity status was scored as follows: 0, alert and playful; 1, alert and playful only when stimulated; 2, alert but not playful when stimulated; 3, neither alert nor playful when stimulated. Body weight was monitored daily. Animals were euthanized when one or more humane endpoint criteria were met: the animal no longer eats or drinks, a decrease in body weight >20% from original weight, presence of severe circulation or respiratory problems, the animal’s behavior and locomotion are severely abnormal, the infection causes severe clinical signs, evidence of mouth breathing or an activity score of 3. Throat and nose swabs were collected every day and were stored at −80°C in transport medium [Hank’s balanced salt solution containing 0.5% of lactalbumin (Sigma-Aldrich, Zwijndrecht, The Netherlands), 10% of glycerol (Sigma-Aldrich), 200 U/mL penicillin, 200 mg/mL streptomycin, 100 U/mL polymixin B sulphate (Sigma-Aldrich), and 250 mg/mL gentamicin (Gibco)] for endpoint titration in MDCK cells. Three animals were euthanized at 3 and 6 dpi by exsanguination under anesthesia to allow comparison with historical experiments, unless for ethical reasons ferrets needed to be euthanized earlier. Tissues harvested for virological examination were homogenized in transport medium using the FastPrep system (MP Biomedicals, Eschwege, Germany) with two one-quarter-inch ceramic sphere balls, centrifuged 1500 × *g* for 10 minutes aliquoted, and stored at −80°C for endpoint titration in MDCK cells. Tissues harvested for histological examination to examine the type of lesions associated with virus presence were fixed in 10% neutral-buffered formalin, embedded in paraffin, sectioned at 4 m, and stained with hematoxylin and eosin (HE) for examination by light microscopy.

### Immunohistochemistry

For detection of influenza A virus antigen, sequential slides of all tissues were stained with a primary antibody against the influenza A NP as described previously (49, 50). In each staining procedure, an isotype control was included as a negative control, and a lung section from a cat infected experimentally with H5N1 was used as positive control (51).

### Deep sequencing analysis

RNA was extracted using a QIAamp viral RNA minikit (catalog number 52904; Qiagen, Venlo, The Netherlands). M-RTPCR amplification was performed with a SuperScript III high-fidelity RT-PCR Kit (catalog number 12574–023; Invitrogen, Leiden, The Netherlands) according to the manufacturer’s instructions using the Opti1 primer set, consisting of primers Opti1-F1 (5′-GTTACGCGCCAGCAAAAGCAGG), Opti1-F2 (5′-GTTACGCGCCAGC**G**AAAGCAGG), and Opti1-R1 (5′-GTTACGCGCCAGTAGAAACAAGG). DNA amplicons were purified using an Agencourt AMPure XP 5 mL Kit (catalog number A63880; Beckman Coulter, Woerden, The Netherlands). Library preparation and fragmentation were done using the Roche KAPA Hyperplus Kit, followed by sequencing on the MiSeq instrument, according to the instructions of the manufacturer (Illumina, Cambridge, UK). Raw sequence data were analyzed and mapped using the CLC Genomics software package, workbench 11 (CLC Bio, Arhus, Denmark). The sequences were trimmed to improve quality using a Phred score of 20. Reads were aligned to reference sequence A/Black-headed gull/Netherlands/29/2017 (EPI1131093-EPI1131100). The threshold for mutation detection was manually set at 1% and 5%, for the virus inoculum and ferret samples, respectively.

### Hemagglutination inhibition assay

Ferret antisera were prepared as previously described (52). Ferret antisera were pre-treated overnight with receptor destroying enzyme (*Vibrio cholerae neuraminidase*, VCNA) at 37°C and incubated at 56°C for 1 h the next day. Twofold serial dilutions of the antisera, starting at a 1:20 dilution, were mixed with 25 µL of a virus stock containing four hemagglutinating units and were incubated at 37°C for 30 minutes. Subsequently, 25 µL of 1% turkey erythrocytes was added, and the mixture was incubated at 4°C for 1 h. Hemagglutination inhibition was read and was expressed as the reciprocal value of the highest dilution of the serum that completely inhibited agglutination of virus and erythrocytes.

### Modified red blood cell assay

Modified TRBCs were prepared as described (53) with slight modifications. Briefly, all sialic acids were removed from the surface of TRBC by incubation of 62.5 mL of 20% TRBC in PBS with 50 mU of VCNA (Roche, Almere, the Netherlands) in 8 mM calcium chloride at 37°C for 1 h. Complete removal of SAs was confirmed by loss of HA of treated TRBC using control viruses. Resialylation was done using 0.5 mU α2,3-(N)-sialyltraferase (Sigma-Aldrich, Zwijndrecht, the Netherlands) or 25 mU α2,6-(N)-sialyltransferase (Sigma-Aldrich) and 1.5 mM CMP-SA (Merck, Darmstadt, Germany) at 37°C in 75 mL for 2 h to produce α2,3-TRBC and α2,6-TRBC, respectively. After washing, the TRBCs were resuspended in PBS containing 1% BSA to a final concentration of 0.5%. Resialylation of either α2,3-SA or α2,6-SA was confirmed by HA using viruses with known receptor specificity. Viruses were tested in standard HA assay using native and resialylated TRBCs. In brief, twofold dilutions of virus were made in PBS. An equal volume of 0.5% TRBCs were added and incubated at 4°C for 1 h before reading the HA titer.

### HA temperature stability assay

Virus solutions were incubated for 30 minutes at different temperatures before performing an HA assay using TRBCs. Twofold dilutions of virus in PBS containing 0.25% red blood cells were prepared in a U-shaped 96-well plates and were incubated for 1 h at 4°C, and agglutination was recorded.

### HA acid stability assay

The open reading frames (coding region) of the HA genes of virus isolates were cloned into the pCAGGS expression vector using Gibson Assembly (New England Biolabs, Ipswich, MA, USA). Fusion was tested as previously described (54) in a cell content mixing assay in which two 10 cm dishes containing Vero-118 cells were transfected with 5 µg of pCAGGS-HA and 5 µg of pEGFP-N1 (as transfection control) using Xtremegene Transfection Reagent (Roche). One day after transfection, cell populations were harvested using trypsin-EDTA and plated in a 12-well plate format. The next morning, cells were exposed to PBS at different pH for 10 minutes. Cells were fixed 24 h after the pH-pulse using 70% ice-cold acetone, washed, and stained using a 20% Giemsa mixture for microscopy (Merck Millipore, Darmstadt, Germany).

### Minigenome assay

The open reading frames (coding region) of the PB2, PB1, PA, and NP genes of virus isolates were cloned into the pPPI4 expression vector (55) using Gibson assembly (New England Biolabs). Substitution of D701N in PB2 was introduced using QuikChange II Site-Directed Mutagenesis Kit (Agilent, Amstelveen, The Netherlands). A plasmid coding for a model viral RNA (vRNA), consisting of the FF luciferase open reading frame flanked by the non-coding regions of segment 8 of an H5N1 influenza A virus under the control of a human pPolI was used for minigenome assays (26). Production of mRNA of this FF luciferase vRNA is solely possible by transcription of the produced vRNA by the influenza virus polymerase complex. Therefore, the FF luciferase activity is a measure of the amount of mRNA produced and thereby of the polymerase complex activity. Transfection of pRL plasmid (Promega, Leiden, The Netherlands), expressing *Renilla* luciferase, served as an internal control to normalize variation in transfection efficiency and sample processing.

Human HEK-293T was seeded 1 day prior to the experiment into 96-well plates. An amount of 25 ng of the FF reporter plasmid, 50 ng of each of the plasmids encoding PB2, PB1, and PA, 100 ng of NP, and 2 ng of the *Renilla* luciferase expression plasmid in 50 μL Opti-MEM (Gibco, Thermo Fisher, Lelystad, The Netherlands) was mixed with 50 μL Optim-mem containing Lipofectamine 2000 (Invitrogen, Thermo Fisher) or TransIT-X2 Transfection Reagent (Mirus Bio LLC, Madison, WI, USA) in a 1:3 ratio and incubated for 20 minutes at room temperature. An amount of 20 μL of the transfection mixture was added to each well. Each transfection was performed in quadruplo in at least two independent experiments. Twenty-four hours post-transfection luminescence was measured using the Dual-Luciferase Reporter Assay System (Promega) using a GloMax Luminometer according to the manufacturer’s instructions (Turner BioSystems, Sunnyvale, CA, USA).
